# Dopamine Cytotoxicity Involves Both Oxidative and Nonoxidative Pathways in SH-SY5Y Cells: Potential Role of Alpha-Synuclein Overexpression and Proteasomal Inhibition in the Etiopathogenesis of Parkinson's Disease

**DOI:** 10.1155/2014/878935

**Published:** 2014-04-02

**Authors:** Kalpita Banerjee, Soumyabrata Munshi, Oishimaya Sen, Vishmadeb Pramanik, Tapasi Roy Mukherjee, Sasanka Chakrabarti

**Affiliations:** ^1^Department of Biochemistry, Institute of Post-Graduate Medical Education & Research, 244B, Acharya J. C. Bose Road, Kolkata 700020, India; ^2^Department of Neuroscience, Burke-Cornell Medical Research Institute, 785 Mamaroneck Avenue, White Plains, NY 10605, USA; ^3^Department of Neuroscience, Rosalind Franklin University of Medicine and Science, 3333 Green Bay Road, North Chicago, IL 60064, USA; ^4^DNA Laboratory, Anthropological Survey of India, 27, J. N. Road, Kolkata 700016, India; ^5^National Institute of Cholera and Enteric Diseases, P-33 C.I.T. Road, Kolkata 700010, India

## Abstract

*Background*. The cytotoxic effects of dopamine (DA) on several catecholaminergic cell lines involve DA oxidation products like reactive oxygen species (ROS) and toxic quinones and have implications in the pathogenesis of sporadic Parkinson's disease (PD). However, many molecular details are yet to be elucidated, and the possible nonoxidative mechanism of dopamine cytotoxicity has not been studied in great detail. *Results*. Cultured SH-SY5Y cells treated with DA (up to 400 *μ*M) or lactacystin (5 *μ*M) or DA (400 *μ*M) plus N-acetylcysteine (NAC, 2.5 mM) for 24 h are processed accordingly to observe the cell viability, mitochondrial dysfunctions, oxidative stress parameters, proteasomal activity, expression of alpha-synuclein gene, and intracellular accumulation of the protein. DA causes mitochondrial dysfunction and extensive loss of cell viability partially inhibited by NAC, potent inhibition of proteasomal activity marginally prevented by NAC, and overexpression with accumulation of intracellular alpha-synuclein partially preventable by NAC. Under similar conditions of incubation, NAC completely prevents enhanced production of ROS and increased formation of quinoprotein adducts in DA-treated SH-SY5Y cells. Separately, proteasomal inhibitor lactacystin causes accumulation of alpha-synuclein as well as mitochondrial dysfunction and cell death. *Conclusions*. DA cytotoxicity includes both oxidative and nonoxidative modes and may involve overexpression and accumulation of alpha-synuclein as well as proteasomal inhibition.

## 1. Introduction

Parkinson's disease (PD), which is a progressive neurodegenerative disorder affecting mainly the elderly people, appears in two major forms, familial and sporadic, and the latter variety accounts for nearly 90–95% of PD subjects. The pathological hallmark of PD is the degeneration of dopaminergic neurons of substantia nigra that project into striatum [1]. The underlying mechanisms of dopaminergic neuronal death in sporadic PD are still uncertain, but mitochondrial dysfunctions, oxidative stress, proteolytic impairment with abnormal accumulation of proteins like alpha-synuclein, and inflammatory reactions are key elements in this complex pathogenesis [1–3]. Several animal and cell-based experimental models have been widely used to elucidate the mechanism of dopaminergic neuronal death in sporadic PD [4–8]. Although in some models various toxins like 6-hydroxydopamine (6-OHDA), rotenone, and 1-methyl-4-phenyl-1,2,3,6-tetrahydropyridine (MPTP) have been used to induce cell death, the cytotoxic action of dopamine (DA) on catecholaminergic cell lines is of special relevance in the context of PD pathogenesis because DA is endogenously available in the body [5, 6, 9, 10]. A large body of evidence has already implicated that DA oxidation products like reactive oxygen species (ROS) and toxic quinones can trigger apoptotic death in cultured PC12 and SH-SY5Y cells in which mitochondrial dysfunction plays a critical role [11–16]. Although most of these studies on DA cytotoxicity have suggested the oxidative mode of cellular damage, a few have also indicated the existence of a nonoxidative pathway in this process [17, 18].

The involvement of proteasomal impairment with abnormal accumulation of proteins, especially alpha-synuclein, in PD pathogenesis has been suggested based on multiple lines of evidence in sporadic and familial PD [19, 20]. A substantial body of evidence also indicates the toxic effects of alpha-synuclein on different cell lines or on isolated brain mitochondria [21–26]. Since proteasomal inactivation and alpha-synuclein accumulation occur in the substantia nigral neurons in PD, it is likely that these phenomena have some unknown links with dopamine. We hypothesize that alpha-synuclein accumulation and proteasomal inactivation may be linked with either the oxidative or nonoxidative mode of DA toxicity, which could be tested in a cell-based model. In the present study, we have, therefore, exposed SH-SY5Y cells (a neuroblastoma cell line with the robust expression of alpha-synuclein) to varying concentrations of DA over a period of 24 h with or without radical and quinone scavengers and measured cytotoxicity parameters as well as alpha-synuclein content and proteasomal activity.

## 2. Materials

The reagents used in the study were obtained from the following sources: Dulbecco's Modified Eagle's Medium/Nutrient F-12 Ham (DMEM/F-12 Ham, 1 : 1), fetal bovine serum (FBS), 5,5′,6,6′-tetrachloro-1,1′,3,3′-tetraethylbenzimidazolocarbocyanine iodide (JC-1), ATP bioluminescent assay kit, penicillin, streptomycin, and amphotericin B from Sigma Aldrich, USA; dopamine (DA), N-acetylcysteine (NAC) and Amplex Red from Acros Organics, USA; fluorogenic peptides (Suc-Leu-Leu-Val-Tyr-AMC and Z-Leu-Leu-Glu-AMC) and lactacystin from Alexis Biochemicals, USA; nitroblue tetrazolium (NBT) from Promega, India; polyclonal rabbit anti-alpha-synuclein antibody from Santa Cruz Biotechnology, USA; horseradish peroxidase- (HRP-) conjugated secondary goat anti-rabbit IgG antibody from Bangalore Genei, India; RNA extraction kit from Roche Applied Science, Germany; and reverse transcription kit, forward and reverse primers for both the target (alpha-synuclein) and the reference (*β*-actin) genes from Roche Diagnostics, Germany. All common chemicals and reagents used were of the highest analytical grade available and were obtained from Sisco Research Laboratories, India.

## 3. Methods

### 3.1. Cell Culture and Treatment Paradigm

Undifferentiated human neuroblastoma SH-SY5Y cells obtained from American Type Culture Collection (ATCC, USA) were grown in DMEM/F-12 Ham containing 10% (v/v) heat-inactivated FBS, 50 units/mL penicillin, 50 *μ*g/mL streptomycin, and 2.5 *μ*g/mL amphotericin B in a humidified environment containing 5% CO_2_ and 95% air at 37°C in 25 cm^2^ sterile tissue culture flasks. SH-SY5Y cells (70–75% confluent) were incubated with or without varying concentrations of DA (100–400 *μ*M) in the absence or presence of other additions like NAC (2.5 mM) or lactacystin (5 *μ*M) for 24 h. Control and treated cells were analyzed for cell death, mitochondrial functions, oxidative stress parameters, proteasomal activity, and alpha-synuclein content and alpha-synuclein gene expression.

### 3.2. Assessment of Cell Death

#### 3.2.1. Trypan Blue Assay

An aliquot of cell suspension in phosphate-buffered saline (PBS) was mixed with an equal volume of trypan blue solution (0.4% in PBS), and the cells were counted in a Neubauer chamber. The cell death was expressed as the percentage of trypan blue positive cells in the total population of stained and unstained cells counted by an observer blind to treatment protocol [27].

#### 3.2.2. Lactate Dehydrogenase (LDH) Assay

Cell death was also measured by LDH assay in which the activity of LDH released in the medium, expressed as the percentage of the total LDH (intracellular plus extracellular), was measured using NADH-based spectrophotometric assay [28].

### 3.3. Measurement of Mitochondrial Membrane Potential in SH-SY5Y Cells

Mitochondrial transmembrane potential was measured in intact SH-SY5Y cells by using a cationic carbocyanine dye JC-1 which remains distributed in the cytosol as monomers while after entering the mitochondria driven by the electrochemical gradient undergoes concentration-dependent aggregation to form J-aggregates. The monomers emit a green fluorescence (*λ*ex 490 nm, *λ*em 530 nm) and J-aggregates a red fluorescence (*λ*ex 490 nm, *λ*em 590 nm) and the ratio of fluorescence intensities at 590 nm to 530 nm indicates mitochondrial transmembrane potential [11]. Briefly, control and treated cells were washed twice in PBS and then incubated in serum-free DMEM for 30 min in the presence of JC-1 (10 *μ*M) at 37°C in the dark. The cells were pelleted down and then washed thrice with serum-free DMEM, suspended with suitable dilutions in the same medium and the fluorescence emission intensities (590 nm and 530 nm) were measured with excitation at 490 nm in a spectrofluorometer (FP-6300, JASCO International Co., Japan).

### 3.4. Measurement of ATP Content in SH-SY5Y Cells

The ATP content was measured in control and treated SH-SY5Y cells by using a commercial kit based on luciferin-luciferase reaction as described previously [11]. Briefly, the treated and control cells were washed with PBS followed by disruption of resuspended cells in 100 *μ*L aliquots by ice-cold lysis-buffer containing 10 mM Tris, 1 mM EDTA, and 0.5% Triton X-100, pH 7.6. They were immediately used for ATP measurement using the ATP-assay mix provided by the kit [11]. Using a microplate luminometer (Biotek, ELX-800, USA), the luminescence generated was measured promptly. Appropriate blank reactions consisting of ATP-assay mix and lysis buffer without cells were performed to measure background luminescence. The intracellular content of ATP was calculated from an ATP standard curve generated by plotting the luminescence readings against known concentrations of ATP (0.08 nmol/mL–40 nmol/mL) and the values were calculated as nmol of ATP per mg of protein.

### 3.5. Assessment of Oxidative Stress Parameters in SH-SY5Y Cells

#### 3.5.1. Measurement of Hydrogen Peroxide (H_2_O_2_) Generation Using Amplex Red

H_2_O_2_ production from SH-SY5Y cells during* in vitro* incubation was measured using the H_2_O_2_-specific fluorescent dye Amplex Red (N-acetyl-3,7-dihydroxyphenoxazine) as described earlier [11, 29]. Briefly, control and treated cells were harvested and washed twice with phosphate-buffered saline (PBS) and finally resuspended in Krebs-Ringer's buffer containing 10 mM glucose (pH 7.4). An aliquot of this cell suspension was incubated in the same Krebs-Ringer's buffer with 50 *μ*M Amplex Red and 1 U/mL horseradish peroxidase for 15 min in the dark at room temperature. At the end of the incubation an aliquot of the incubation mixture was appropriately diluted and the fluorescence emission was measured at an excitation wavelength of 530 nm and an emission wavelength of 590 nm using a spectrofluorometer (FP6300, JASCO International Co., Japan). The background fluorescence was subtracted from the sample fluorescence using a blank containing the reaction mixture without any added cells. Fluorescence intensity was converted to nmol of H_2_O_2_ produced per mg of protein using a calibration curve utilizing pure H_2_O_2_ in the concentration range of 50 nM to 400 nM.

#### 3.5.2. Nitroblue Tetrazolium (NBT)/Glycinate Assay

Quinoprotein adduct formation in SH-SY5Y cells was measured by the NBT/glycinate assay following published procedures [30, 31]. Briefly, control and treated cells were first washed with phosphate-buffered saline and then suspended in 50 mM phosphate buffer (pH 7.4) in a total volume of 200 *μ*L, followed by addition of 200 *μ*L of 10% trichloroacetic acid for protein precipitation. The precipitate was washed twice with ethanol, treated with chloroform-methanol (2 : 1, v/v), and vortexed thoroughly. It was then centrifuged at 5000 g for 10 min and the supernatant was removed. To the delipidated protein precipitate 1 mL of NBT reagent (0.24 mM NBT in 2 M potassium glycinate, pH 10) was added followed by incubation in the dark for 1 h on a shaker. The blue-purple color developed in the reaction mixture was suitably diluted and absorbance taken at 530 nm using a spectrophotometer (model 117, Systronics, India).

### 3.6. Proteasomal Degradation Assay

Proteasome activity was assayed in the cell lysate by measuring the release of 7-amino-4-methylcoumarin (AMC) from the fluorogenic peptides Suc-Leu-Leu-Val-Tyr-AMC and Z-Leu-Leu-Glu-AMC. Cells were lysed in 20 mM Tris-HCl, 1 mM EDTA buffer, pH 7.5 by 4 cycles of freezing-thawing followed by centrifugation at 5000 rpm for 10 min. The supernatant was collected and an aliquot (100–300 *μ*g protein) was added to the assay mixture in a total volume of 300 *μ*L containing 50 *μ*M Z-Leu-Leu-Glu-AMC, 50 *μ*M Suc-Leu-Leu-Val-Tyr-AMC, 5 mM ATP, and 5 mM Mg acetate in Tris-EDTA buffer with or without lactacystin (0.1 *μ*M). The assay mix was incubated at 37°C for 30 min followed by the fluorescence measurement at *λ*ex 360 nm and *λ*em 465 nm. The assay produced proportional response up to 300 *μ*g of protein. Lactacystin sensitive activity was taken as the proteasomal activity and expressed as arbitrary fluorescence units/100 *μ*g of protein [20].

### 3.7. Immunodetection of Alpha-Synuclein in SH-SY5Y Cells

SH-SY5Y cells were lysed in cell lysis buffer (20 mM Tris, 1 mM EDTA, pH 7.4) by repeated freeze-thaw. The lysate was then centrifuged for 10 min at 12,000 rpm at 4°C. The supernatant was subjected to 12% SDS-PAGE followed by immunoblotting utilizing standard blotting protocols and using primary antibody (polyclonal rabbit anti-alpha-synuclein antibody, 1 : 1000 dilution) and HRP-conjugated secondary goat anti-rabbit IgG antibody (1 : 5000 dilution). Finally, the blot was developed by the enhanced chemiluminescence technique. Equal amount of protein was loaded in each well for SDS-PAGE. The band intensities were measured using a gel doc apparatus (G-Box, Syngene, UK) and analyzed with the help of the software Syngene Gene Tools (File version: 4.01.04) using gamma-actin loading control normalization.

### 3.8. Quantitative RT-PCR for Alpha-Synuclein Gene Expression

Total RNA was extracted from control and treated cells using a commercial kit (Roche Applied Science, Germany) and following the manufacturer's protocol. Reverse transcription was performed as per the manufacturer's protocol (Roche Diagnostics, Germany) from total RNA utilizing random primers. The real-time PCR analysis of the cDNA samples for a fragment (187 bp) of alpha-synuclein gene was performed in triplicate using SYBR Green in a reaction volume of 20 *μ*L using 10 pmol each of forward and reverse primers for both the target (alpha-synuclein) and the reference *β*-actin genes (Applied Biosystems, model 7500). The following primers were used: *β*-actin forward—tcaccatggatgatgatatcgcc, *β*-actin reverse—ccacacgcagctcattgtagaagg, alpha-synuclein forward—aggactttcaaaggccaagg, and alpha-synuclein reverse—tcctccaacatttgtcacttgc [32]. The changes in alpha-synuclein gene expression in different samples were calculated from the Ct (threshold cycle) values of the target gene and the reference gene by the relative quantitation method [32].

### 3.9. Protein Estimation

The protein was estimated by Lowry's method after solubilizing the samples in 1% SDS [33].

### 3.10. Statistical Analysis

The statistical analysis of different parameters among 3 or more groups was performed by analysis of variance (ANOVA) followed by post hoc comparison by means of Newman-Keuls test when the *P* value was significant. For the densitometric analysis, Tukey's post hoc test was done. The statistical comparison of different parameters between 2 groups was performed by Student's *t*-test, paired. The *P* value less than 0.05 was considered significant. Each experiment was performed several times and the values were expressed as the mean ± SD (standard deviation) of the number of observations [34].

## 4. Results

### 4.1. DA Effects on Viability of SH-SY5Y Cells

**Figure 1:**
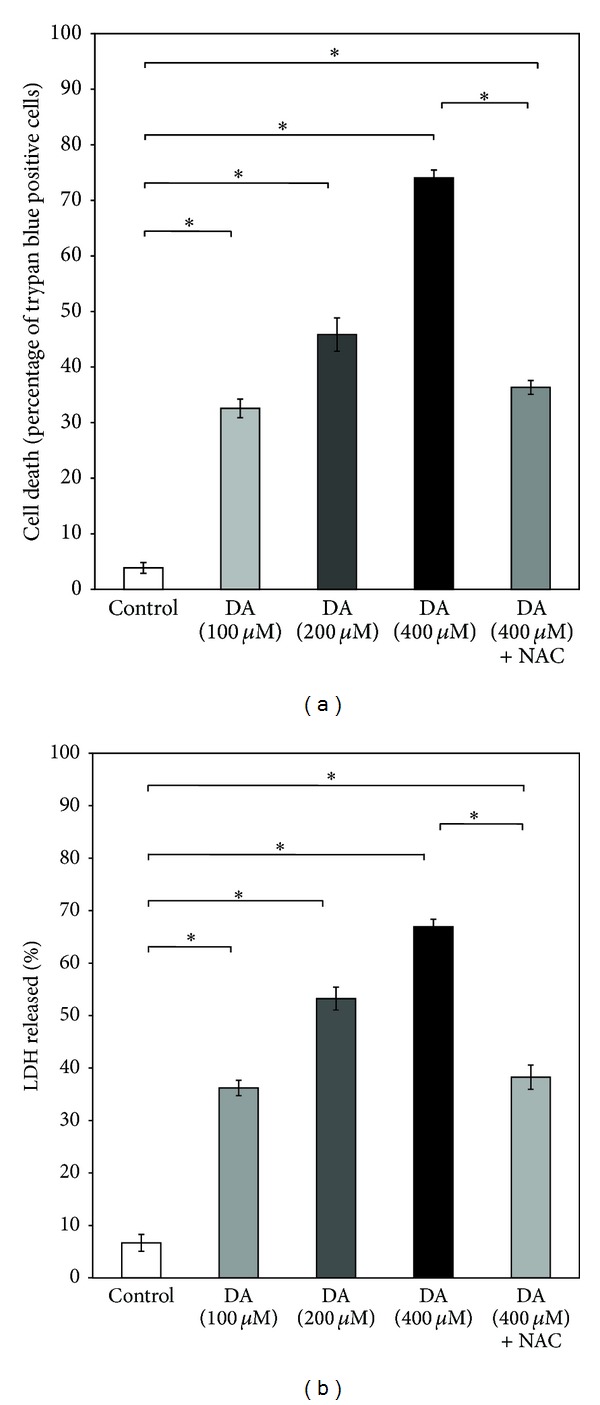
Dose-dependent effects of DA on SH-SY5Y cell-viability. SH-SY5Y cells were treated without (control) or with varying concentrations of DA (100–400 *μ*M) for 24 h in the presence or absence of NAC (2.5 mM), and the cell viability was measured by (a) trypan blue assay or (b) estimation of LDH activity, as described in Section 3. Values are the means ± SD of six observations. Statistically significant differences in cell death among the groups exist at *P* < 0.001 as shown by asterisks (∗) (in (a), *F*  (4,25) = 1177.09, *P* < 0.001; in (b), *F*  (4,25) = 898.99. *P* < 0.001).

When SH-SY5Y cells were exposed to DA, a dose-dependent loss of cell viability was noted as measured by trypan blue exclusion assay (Figure 1(a)). The release of the cytosolic enzyme LDH in the medium reflecting cell death also increased significantly and in a dose-dependent manner after DA exposure of SH-SY5Y cells (Figure 1(b)). After 24 h of exposure to 400 *μ*M DA, a significant loss of cell viability was noted in SH-SY5Y cells, which was prevented to the extent of nearly 50% by concomitant presence of N-acetylcysteine (2.5 mM) in the medium (Figures 1(a) and 1(b)). N-acetylcysteine (2.5 mM) alone did not cause any significant effect on the cell viability compared to control (data not shown).

### 4.2. Effect of DA on Mitochondrial Function in SH-SY5Y Cells

**Figure 2:**
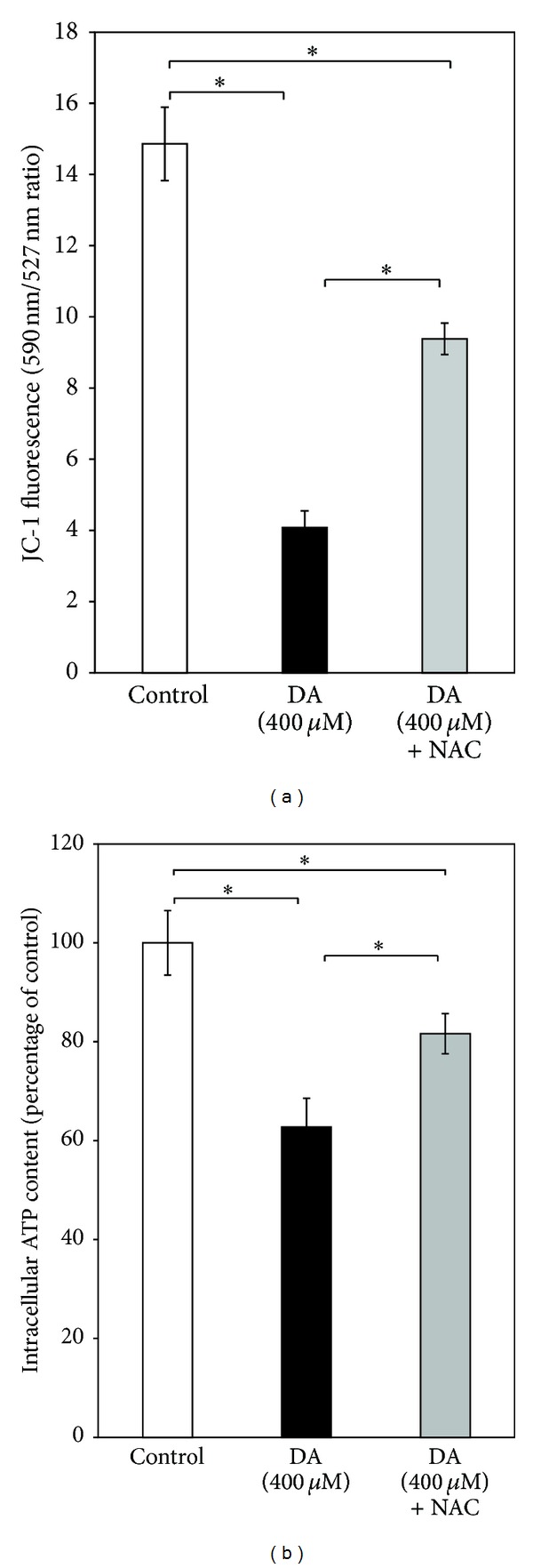
Effect of DA on mitochondrial functions in SH-SY5Y cells. SH-SY5Y cells were incubated without or with DA (400 *μ*M) for 24 h in the absence or presence of NAC (2.5 mM) and (a) mitochondrial membrane potential and (b) intracellular ATP content were measured as described in Section 3. Values are the means ± SD of six observations. Statistically significant differences (*P* < 0.001) exist among the groups as shown by asterisks (∗) (in (a), *F*  (2,15) = 353.56, *P* < 0.001; in (b), *F*  (2,15) = 67.39, *P* < 0.001).

DA (400 *μ*M) for 24 h caused a striking loss of mitochondrial membrane potential in SH-SY5Y cells as evident from a significant (nearly 70%) decrease in the ratio of fluorescence intensities of JC-1 at 590 nm and 527 nm (Figure 2(a)). DA treatment for the same period also decreased the ATP content of SH-SY5Y cells by nearly 40% as compared to control (Figure 2(b)). Both phenomena were prevented partially but significantly by concomitant presence of N-acetylcysteine (2.5 mM) in the medium.

### 4.3. Oxidative Stress Parameters in SH-SY5Y Cells after DA (400 *μ*M) Exposure

**Figure 3:**
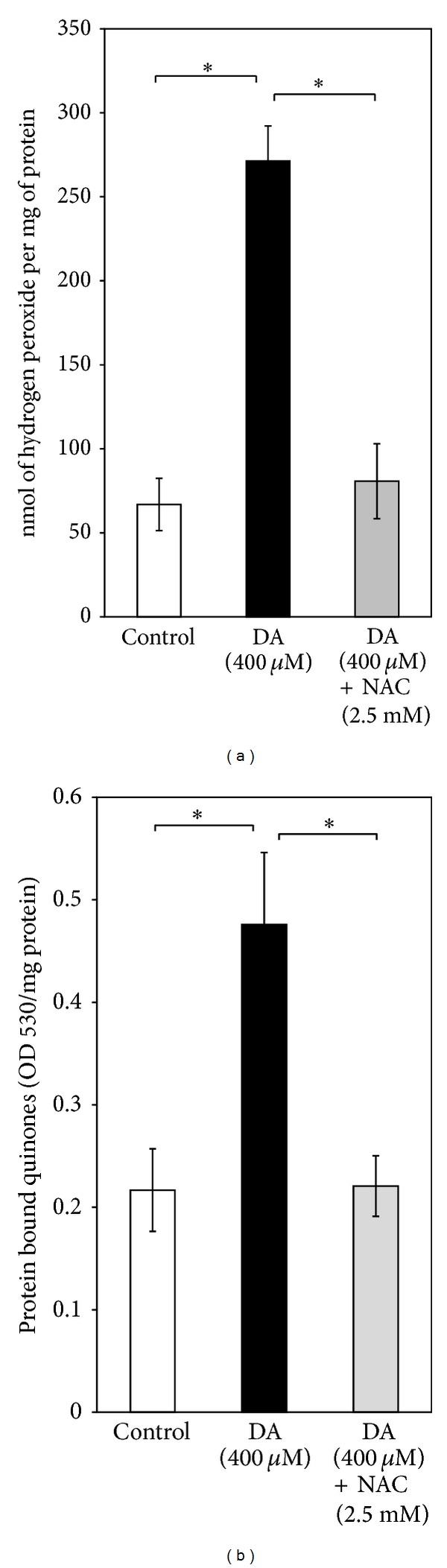
Measurement of oxidative stress parameters. (a) Effect of NAC (2.5 mM) on DA (400 *μ*M)-induced production of hydrogen peroxide (H_2_O_2_) in SH-SY5Y cells: production of H_2_O_2 _was measured during* in vitro* incubation of SH-SY5Y cells (control, 400 *μ*M DA treated and 400 *μ*M DA + 2.5 mM NAC treated) in Krebs-Ringer's buffer as detailed in Section 3. Values (expressed as nmol of H_2_O_2 _per mg protein) are the means ± SD of four observations. Statistically significant differences exist (*F*  (2,9) = 132.63, *P* < 0.001)) between the DA-treated groups and control, as well as between the DA-treated groups and NAC-treated groups, as shown by asterisks (∗). (b) Measurement of protein bound quinones in SH-SY5Y cells: levels of protein bound quinones were measured in SH-SY5Y cells incubated without or with DA (400 *μ*M) for 24 h in the presence or absence of NAC (2.5 mM) as detailed in Section 3. Values (optical density at 530 nm (OD 530) per mg protein) are the means ± SD of four observations. Statistically significant differences exist (*F*  (2,9) = 35.79, *P* < 0.001) between the DA-treated groups and control, as well as between the DA-treated groups and NAC-treated groups, as shown by asterisks (∗).

Following DA (400 *μ*M) exposure for 24 h, a significant increase of ROS production was observed in SH-SY5Y cells, but the phenomenon was completely prevented in cells co-treated with DA (400 *μ*M) and NAC (2.5 mM) (Figure 3(a)).

DA (400 *μ*M)-derived quinones reacted with cellular nucleophiles to produce stable quinoprotein adducts. After DA (400 *μ*M) treatment for 24 h, the quinoprotein adduct formation was increased by twofolds in SH-SY5Y cells which was completely reversed by concomitant treatment with NAC (2.5 mM) (Figure 3(b)).

### 4.4. Proteasomal Inhibition in SH-SY5Y Cells: DA and Lactacystin Effects

**Figure 4:**
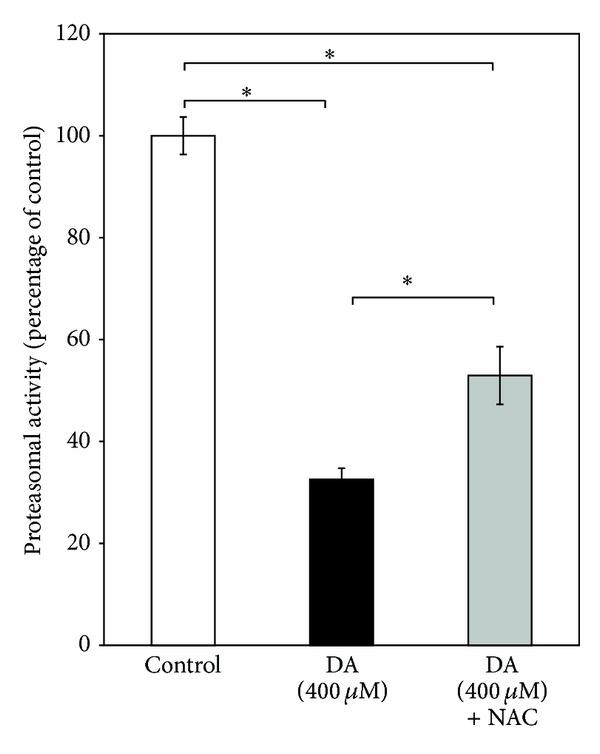
Effect of DA on proteasomal activity in SH-SY5Y cells. Proteasomal activity was measured in SH-SY5Y cells incubated without or with DA (400 *μ*M) for 24 h in the presence or absence of NAC (2.5 mM) as detailed in Section 3. Values (expressed as percentage of the control) are the means ± SD of five observations. Statistically significant differences exist (*F*  (2,12) = 356.38, *P* < 0.001) between the groups as shown by asterisks (∗).

Proteasomal activity in SH-SY5Y cells was decreased by approximately 65% compared to that of control after exposure to DA (400 *μ*M) for 24 h, and in the presence of NAC (2.5 mM) in the culture medium, DA still produced nearly 50% inhibition of the enzyme activity (Figure 4).

**Figure 5:**
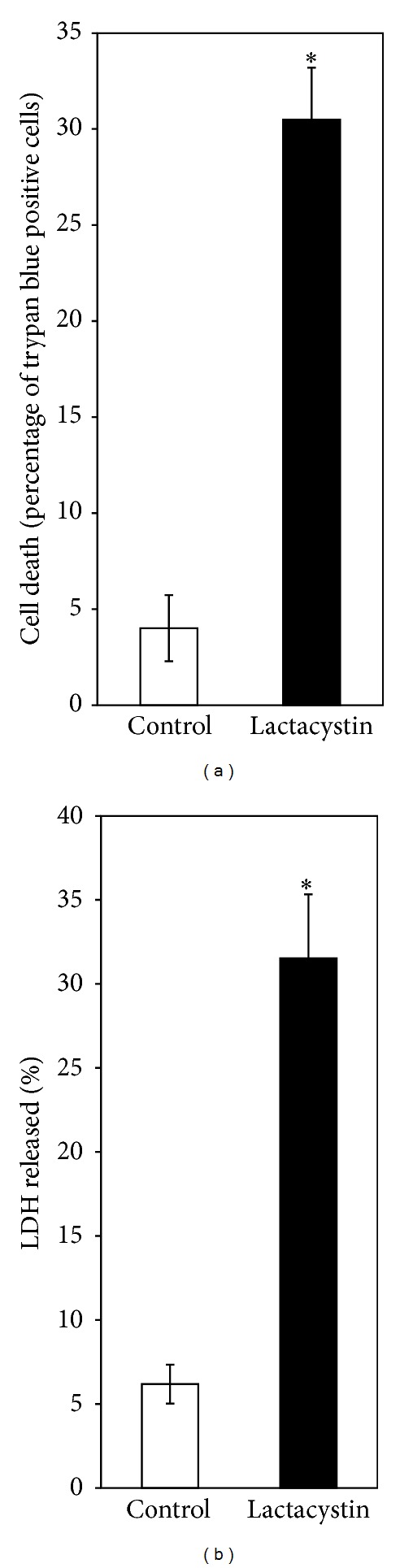
Effect of lactacystin on SH-SY5Y cell-viability. SH-SY5Y cells were treated without (control) or with lactacystin (5 *μ*M) for 24 h followed by the measurement of cell viability by (a) trypan blue method and (b) activity of released LDH, as described in Section 3. Values are the means ± SD of six observations. Student's *t*-test, paired, shows significant difference (*P* < 0.001) in the cell viability between the treated group (M = 30.49, SD = 2.69) and the control group (M = 4.01, SD = 1.72); *t* (5) = 24.23, as shown by asterisks (∗).

**Figure 6:**
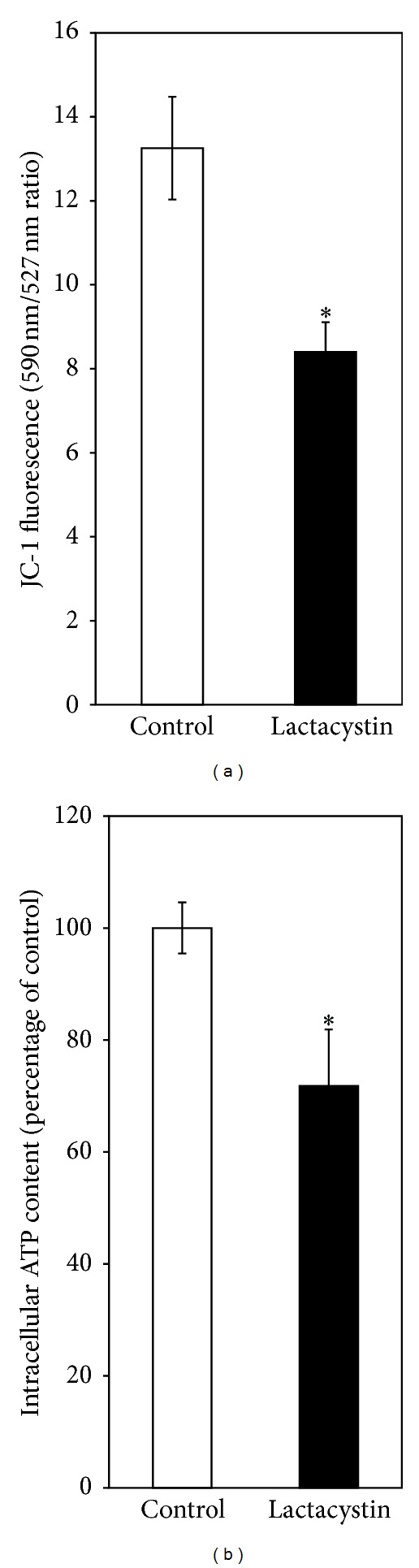
Lactacystin effects on mitochondrial functions in SH-SY5Y cells. SH-SY5Y cells were exposed to lactacystin (5 *μ*M) for 24 h followed by the measurement of (a) mitochondrial membrane potential and (b) intracellular ATP content, as described in Section 3. Values are the means ± SD of six observations. Student's *t*-test, paired, shows significant difference (*P < 0.001) in the mitochondrial membrane potential between the lactacystin-treated group (M = 8.39, SD = 0.71) and the control group (M = 13.25, SD = 1.22); *t*(5) = 12.81 (a). Similarly, a significant difference (*P < 0.01) in the ATP content between the lactacystin-treated group (M = 71.79, SD = 10.08) and the control group exists; *t* (5) = 3.79 (b).

When SH-SY5Y cells were incubated with the proteasomal inhibitor lactacystin (5 *μ*M) for 24 h, cell viability, measured by trypan blue assay and released LDH activity, was significantly reduced (Figures 5(a) and 5(b)). Lactacystin (5 *μ*M) for 24 h also caused a striking loss of mitochondrial membrane potential in SH-SY5Y cells as evident from a significant (nearly 40%) decrease in the ratio of fluorescence intensities of JC-1 at 590 nm and 527 nm (Figure 6(a)). Further, lactacystin (5 *μ*M) treatment for 24 h decreased the ATP content of SH-SY5Y cells by nearly 25% as compared to control (Figure 6(b)).

### 4.5. Alpha-Synuclein Accumulation in SH-SY5Y Cells after Exposure to DA or Lactacystin

**Figure 7:**
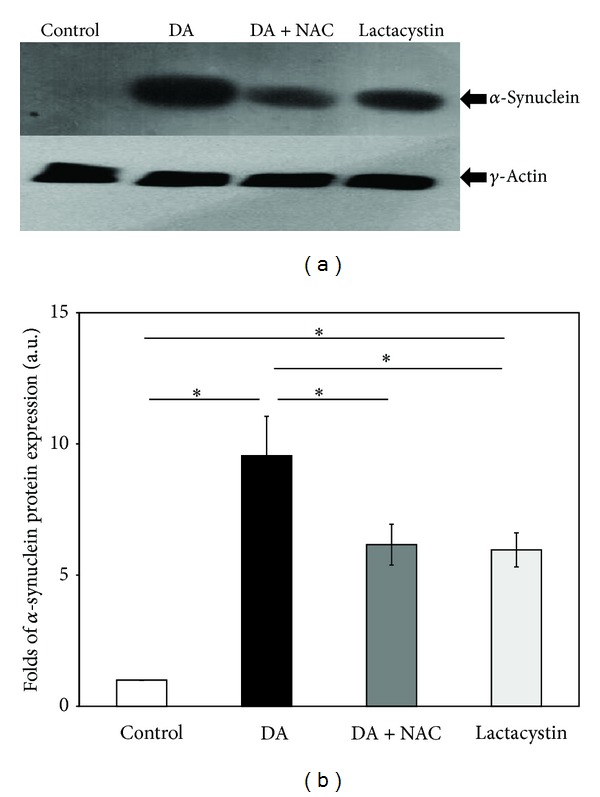
Accumulation of *α*-synuclein in SH-SY5Y cells after DA and lactacystin exposure. SH-SY5Y cells were incubated alone (control), with DA (400 *μ*M), with lactacystin (5 *μ*M), and with 400 *μ*M DA co-treated with 2.5 mM NAC for 24 h. After 24 h of incubation, cell lysates were prepared and processed for Western blotting, as described in Section 3. The specificity of the commercially available antibody has been checked against pure recombinant human *α*-synuclein by Western blotting and immunodot blotting (data not shown). (a) The immunoblot is representative of a set of identical experiments repeated four times. (b) Densitometric analysis of the *α*-synuclein bands obtained by the Western blot (∗ denotes *P* < 0.001 between the groups).

When SH-SY5Y cells were exposed to DA (400 *μ*M) or lactacystin (5 *μ*M) for 24 h, a marked increase in alpha-synuclein content was observed in either case compared to control using the Western immunoblotting technique and densitometric analysis (Figures 7(a) and 7(b)). When the cells were co-treated with DA (400 *μ*M) and NAC (2.5 mM) for 24 h, DA-induced increase in alpha-synuclein content was significantly prevented (Figures 7(a) and 7(b)).

### 4.6. Alpha-Synuclein Gene Expression in SH-SY5Y Cells after Exposure to DA

**Figure 8:**
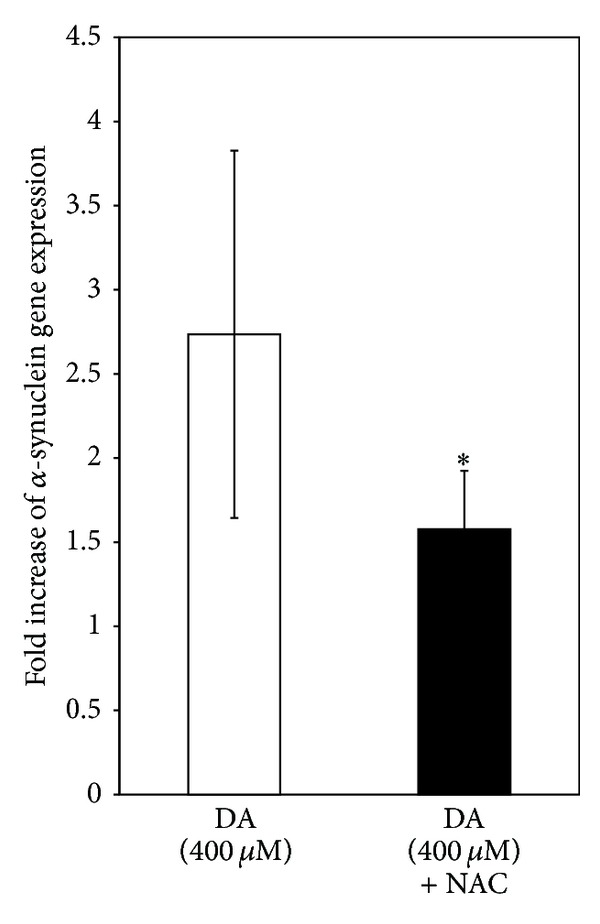
DA effects on *α*-synuclein gene expression in SH-SY5Y cells. SH-SY5Y cells were incubated alone (control) or with DA (400 *μ*M) or 400 *μ*M DA plus 2.5 mM NAC for 24 h. Cellular RNA was isolated and quantitative RT-PCR was carried out for *α*-synuclein gene as detailed in Section 3. Results expressed as the fold increase with respect to the control are the means ± SD of six observations. Statistical significance collected by Student's *t*-test, paired, shows significant difference (*P < 0.05) in the fold of alpha-synuclein gene expression between the DA-treated group (M = 2.73, SD = 1.09) and the NAC co-treated group (M = 1.57, SD = 0.34); *t* (5) = 3.05.

The expression of alpha-synuclein gene in SH-SY5Y cells after exposure to DA (400 *μ*M) for 24 h was increased by 2.7-folds as measured by quantitative RT-PCR, while only 1.5-fold increase was seen when the cells were treated with DA (400 *μ*M) in the presence of 2.5 mM NAC for the same period (Figure 8).

## 5. Discussion

DA- or L-3,4-dihydroxyphenylalanine- (L-DOPA-) induced cell death has been widely studied in several catecholaminergic cell lines or primary neuronal culture from embryonic brain and these are considered to be useful models to study PD pathogenesis [14, 18, 35, 36]. Both ROS and toxic quinones have been implicated in DA-induced cell death in such models which can be effectively prevented by NAC, ascorbate, and other antioxidants suggesting an underlying oxidative mechanism of DA toxicity [14, 35–37]. In a recently published paper, we have also shown that DA induces cell death and mitochondrial dysfunctions in PC12 cells which are mediated by DA-derived quinones, and the phenomenon is nearly completely prevented by NAC which apart from being an antioxidant is also a potent scavenger of toxic quinones [11]. In the present study a dose-dependent death of cultured SH-SY5Y cells with dopamine is observed along with a significant mitochondrial functional impairment, which surprisingly enough are only partially preventable by NAC (2.5 mM) (Figures 1 and 2). This concentration of NAC, however, completely prevents the enhanced ROS production and increased quinoprotein adduct formation in DA-treated SH-SY5Y cells (Figures 3(a) and 3(b)). We used more than sixfold higher concentration of NAC as compared to DA to completely neutralize DA-derived ROS and toxic quinones. Apart from directly scavenging ROS and active quinones, NAC can also upregulate intracellular glutathione (GSH) adding to its antioxidant efficacy. Thus, the results (Figures 3(a) and 3(b)) suggest that DA cytotoxicity under our experimental conditions is mediated partially by nonoxidative mechanisms, which is in contrast to our findings in PC12 cells [11].

### 5.1. Proteasomal Inhibition and DA Cytotoxicity

In our present study, we have tried to explore if the complex mechanism(s) underlying DA cytotoxicity could be related to DA-induced proteasomal inhibition in SH-SY5Y cells. It is observed that the proteasomal inhibition is only mildly attenuated by treatment with a known antioxidant and quinone scavenger NAC (Figure 4) suggesting a primary nonoxidative mechanism of inhibition caused by DA. At this stage we hypothesized that proteasomal inhibition (caused by DA) accounts for the nonoxidative cytotoxic actions of DA such as mitochondrial depolarization and cell death observed during co-treatment with the antioxidant quinone scavenger NAC (Figures 1 and 2). In support of the possibility that proteasomal inhibition may lead to mitochondrial dysfunction and cell death, we have used a “proteasomal-inhibition model” using a well-known proteasomal inhibitor lactacystin to examine the effects on the viability and mitochondrial functions of SH-SY5Y cells. Our results confirm that proteasomal inhibition indeed causes a marked loss of cell viability with mitochondrial membrane depolarization and a decreased intracellular ATP content (Figures 5 and 6), thus validating our hypothesis.

The DA-induced proteasomal inhibition, observed in this study, appears quite unusual because of the failure of NAC, a strong antioxidant and quinone scavenger, to prevent the process. In many earlier studies, the oxidation products of DA such as toxic quinones have been shown to inhibit several enzymes like tyrosine hydroxylase, tryptophan hydroxylase, and Na^+^, K^+^ ATPase either in purified preparation or cell lysate or crude brain membrane fractions [38–40]. Under the present experimental conditions, a direct effect of DA downregulating the expression of proteasomal subunits appears as a distinct possibility.

### 5.2. Alpha-Synuclein in Cytotoxicity of DA

An earlier published study from our laboratory with isolated rat brain mitochondria has shown that alpha-synuclein interferes with mitochondrial bioenergetic functions which have implications in pathogenesis of PD [25]. Hence in our present study we have attempted to find a possible link of DA cytotoxicity with alpha-synuclein. Interestingly enough, we have noticed that DA causes an increase in the alpha-synuclein content within SH-SY5Y cells (Figure 7), by enhancing gene expression (Figure 8) and presumably also by inhibiting proteasomal degradation of alpha-synuclein (Figure 4). The fact that lactacystin treatment of SH-SY5Y cells leads to the intracellular accumulation of alpha-synuclein (Figure 7) confirms that the proteasomal degradation is an important mechanism of alpha-synuclein clearance. It is further observed that DA-induced alpha-synuclein overexpression is only partially prevented by NAC, whereas proteasomal inhibition is prevented even to a lesser extent (Figure 7). As a consequence when SH-SY5Y cells are exposed to DA, a significant accumulation of alpha-synuclein occurs even in the presence of NAC (Figure 7). The accumulated alpha-synuclein in DA and NAC co-treated cells could contribute to mitochondrial dysfunctions and eventual cell death, and this mechanism may be considered as a possible nonoxidative pathway of DA cytotoxicity. In the absence of NAC co-treatment, alpha-synuclein expression and accumulation is even more pronounced (Figures 7 and 8) and would contribute to DA cytotoxicity in a larger measure.

The underlying mechanism of alpha-synuclein overexpression by DA is not evident from this study, except for the fact that the phenomenon is partly mediated by DA oxidation products and partly by some nonoxidative pathway. However, other studies have indicated that DA causes enhancement of alpha-synuclein gene expression by involving stress activated protein kinase, JNK, and p38 or by upregulating the expression of C/EBP beta, an enhancer-binding protein, that regulates the expression of alpha-synuclein [41]. On the other hand, another study has suggested that DA by decreasing the methylation of CpG islands causes an upregulation of alpha-synuclein gene in several cell lines including SH-SY5Y and HEK293 cells [42].

### 5.3. Cytotoxicity of Alpha-Synuclein: Corroboration with Earlier Studies

Our present results implying that alpha-synuclein accumulation could be toxic to SH-SY5Y cells are in agreement with other studies where exogenously added alpha-synuclein or overexpression of alpha-synuclein has led to both mitochondrial impairment and cell death in various cultured cell lines [23, 42, 43]. Further, another study has shown that downregulation of alpha-synuclein gene expression occurs in HEK293 cells through adeno-associated virus (AAV) mediated delivery of alpha-synuclein ribozyme (SynRz) and the injection of the same AAV vector carrying SynRz in to substantia nigra of 1-methyl-4-phenylpyridinium (MPP+) treated rats prevents the apoptotic death of the nigral dopaminergic cells implying the toxic potential of elevated intracellular alpha-synuclein [44]. As previously mentioned, the toxic potential of alpha-synuclein on isolated rat brain mitochondria has also been shown in an earlier work in our laboratory [25]. However, there are scattered reports which have indicated a protective action of alpha-synuclein against oxidative injury in neural cells [45, 46]. The dominant view, however, tends to support the cytotoxic potential of alpha-synuclein [47].

## 6. Conclusions

**Figure 9:**
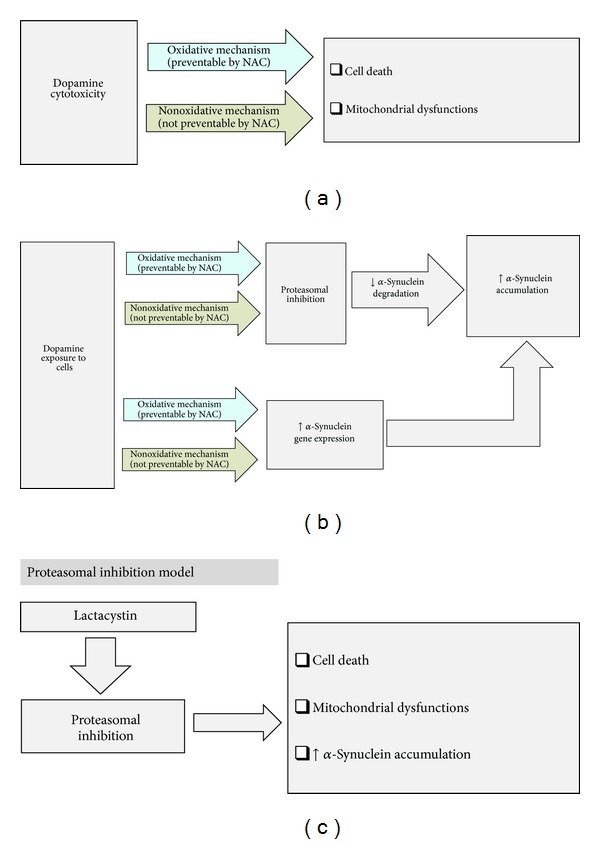
Probable mechanisms of dopamine cytotoxicity in SH-SY5Y cells: involvement of alpha-synuclein and proteasomal inhibition. (a) shows that dopamine causes cell death and mitochondrial dysfunctions involving both the oxidative (NAC responsive) and nonoxidative (NAC nonresponsive) mechanisms. (b) shows that dopamine causes proteasomal inhibition and increased alpha-synuclein gene expression involving similar kind of oxidative (NAC responsive) and nonoxidative (NAC nonresponsive) mechanisms. Both of these effects cause increased intracellular accumulation of alpha-synuclein. (c) shows our “proteasomal inhibition model” using lactacystin resulting in cell death, mitochondrial dysfunctions, and increased intracellular alpha-synuclein accumulation.

Our study has highlighted some important aspects of DA cytotoxicity, especially the nonoxidative mode, in a cell-based model, and suggested that proteasomal inactivation and alpha-synuclein accumulation are potential contributors to this process.  Figure 9 provides a schematic diagram of the underlying mechanisms involved in dopamine cytotoxicity as evident from this paper. It remains unclear if these phenomena are relevant in the actual pathogenesis of PD, but several findings are quite suggestive in this regard. For example, a crucial evidence is the postmortem finding showing proteasomal inactivation in substantia nigra of PD patients with selective loss of 20/26S proteasomal subunits [19]. Further, decreased methylation of intron 1 of alpha-synuclein gene leading to overexpression of this gene has been reported in substantia nigra of postmortem brain of PD patients [42]. The alpha-synuclein content of normal brain is around 1 *μ*M, while in postmortem PD brain the level reported is much higher [48–50]. Moreover, alpha-synuclein is the predominant protein component of Lewy bodies which appear in the degenerating nigral neurons in the brain of PD patients [3, 51]. The genome-wide association studies have also implicated alpha-synuclein gene with PD pathogenesis [52]. All these facts emphasize the need for a more detailed study on proteasomal inactivation, alpha-synuclein overexpression, and accumulation and toxicity in dopaminergic neuronal death in PD, and DA-based cytotoxicity model, as used here, could be very important for further elaborate studies.

## Abbreviations

6-OHDA: 6-Hydroxydopamine
AMC: 7-Amino-4-methylcoumarin
DA: Dopamine
DMEM/F-12 Ham: Dulbecco's Modified Eagle's Medium/Nutrient F-12 Ham
FBS: Fetal bovine serum
HRP: Horseradish peroxidase
JC-1: 5,5′,6,6′-Tetrachloro-1,1′,3,3′-tetraethylbenzimidazolocarbocyanine iodide
JNK: c-Jun amino-terminal kinases
L-DOPA: L-3,4-Dihydroxyphenylalanine
MPP+: 1-Methyl-4-phenylpyridinium
MPTP: 1-Methyl-4-phenyl-1,2,3,6-tetrahydropyridine
NAC: N-Acetylcysteine
NBT: Nitroblue tetrazolium
PD: Parkinson's disease
ROS: Reactive oxygen species.
